# Evidence for a Low Number Prior in Children's Intuitive Number Sense

**DOI:** 10.1111/cdev.14231

**Published:** 2025-04-04

**Authors:** Miranda N. Long, Darko Odic

**Affiliations:** ^1^ Department of Psychology University of British Columbia Vancouver British Columbia Canada

**Keywords:** ANS, Bayesian inference, Bayesian priors, development, natural scene statistics, number perception

## Abstract

Children rely on their Approximate Number System to intuitively perceive number. Such adaptations often exhibit sensitivity to real‐world statistics. This study investigates a potential manifestation of the ANS's sensitivity to real‐world statistics: a negative power‐law distribution of objects in natural scenes should be reflected in children's expectations about number, or in more Bayesian terms, a low number prior distribution. Five‐ to eight‐year‐old children (*n* = 80; 39 girls, 41 boys) and adults (*n* = 20) in 2022 completed a number discrimination task in which one side was corrupted by perceptual noise. Children and adults demonstrate a low number prior. No age‐related differences were observed, suggesting that the prior is formed by age five and does not strengthen with age.

From birth, children can intuitively represent how many objects are in a visual scene (Izard et al. 2009; Feigenson et al. 2004; Odic and Starr 2018; Xu and Spelke 2000). This ability is not trivial, as it relies on first identifying the relevant countable objects and then abstracting away the non‐numeric features, such as size, shape, location, etc. (DeWind et al. 2015; Dramkin et al. 2022; Paul et al. 2022). Understanding the structure and function of the intuitive number sense—i.e., the Approximate Number System (ANS)—is of widespread interest in developmental psychology, because it shows how children's perception can encode abstract properties and because of its ties to other cognitive abilities (Libertus et al. 2020; Park and Brannon 2013).

Researchers have recently focused on the ANS's sensitivity to information and problems that occur in real‐world contexts (Odic and Oppenheimer 2023; Sanford and Halberda 2024; Testolin et al. 2020). Beyond helping us understand the utility of the ANS in naturalistic scenarios, this work is further informed by the argument that the ANS emerged through evolutionary pressures (Brannon and Terrace 2002). Consistent with this, the ANS is found in many non‐human animals (Cantlon and Brannon 2007; Howard et al. 2019), including from birth (Rugani et al. 2015), and has been shown to inform decisions in naturalistic settings (e.g., monkeys appear to use their ANS when migrating; Piantadosi and Cantlon 2017).

One prominent statistical feature of numbers in the real world is the *negative power‐law distribution of numbers* (Piantadosi 2016; Testolin et al. 2020). When examining distributions of objects in the real world, collections of objects with a smaller cardinality (i.e., 1–10) are more likely to occur than collections of objects with a larger cardinality. Analysis of real‐world photographs, for example, shows that scenes containing five items are more than 47 times more likely than scenes containing fifteen items (Testolin et al. 2020). A perceptual system optimized to function in the real world should prioritize reliable mappings for things that occur most often in the real world (Gold and Stocker 2017; Loh and Bartulovic 2014). Piantadosi (2016) theorizes that this distributional property explains why the ANS represents smaller numbers with higher precision than larger numbers, resulting in scalar variability and Weber's law (Feigenson et al. 2004; Odic and Starr 2018).

In the current work, we are interested in another behavior predicted by the ANS's theorized adaptation to real‐world statistics. A power‐law distribution of number—puts pressures on which values should be coded with higher precision (Piantadosi 2016)—might also be reflected in children's *expectations* about number or, in more Bayesian terms, their *priors* (Seriès and Seitz 2013; Gold and Stocker 2017). Perception is often described in terms of Bayesian inference, with immediate sensory information (the *likelihood*) combined with priors to form the most probable picture of what is in our environment (de Lange et al. 2018; Gold and Stocker 2017; Girshick et al. 2011; Seriès and Seitz 2013). Priors often reflect the statistical patterns in our environment, shaped by evolutionary pressures or learned via experience, to optimize the use of our limited neural resources to efficiently navigate our world (Gold and Stocker 2017; Loh and Bartulovic 2014). This is particularly useful when sensory information alone is ambiguous or unreliable. For example, if trying to estimate the number of ducks in a pond on a foggy day, our knowledge about the typical number of ducks in a pond can lead to a more accurate estimate.

This duck‐estimation example reflects the common approach for testing priors in the lab. Given that our response is a combination of both the immediate sensory information and our priors, sensory input can be corrupted by embedding increasing levels of perceptual noise into the experimental task, preventing the sensory information alone from being as informative and thus revealing the prior (de Lange et al. 2018; Gold and Stocker 2017). When relying on priors, participants' responses are often biased in the direction of the mean of the prior, especially on trials where the stimulus (external) information is perceptually noisier. There are instances where participants' responses are biased *away* from a prior, but this is when sensory (internal) noise is used (Wei and Stocker 2015). The use of stimulus noise has been used to demonstrate the existence of priors that truly reflect properties of real‐world statistics in many domains, including speed perception (Stocker and Simoncelli 2006).

In our study, children and adults were shown a number discrimination task in which they decided which of two sides has more objects. One side was always embedded in perceptual noise. If participants possess a low number prior, we would expect that the “noisy” side would be consistently underestimated, reflecting the pull of the unreliable sensory information toward the mean of the low number prior. If participants do not have such a prior, however, then the “noisy” side would result in more imprecise guessing, showing no consistent pull toward low numbers. As our noise manipulation, we chose contrast, which has been previously used in number perception research as a method of stimulus noise (Cheyette and Piantadosi 2020). Previous work has also shown that contrast manipulation sometimes results in *overestimation* of number (Lei and Reeves 2023), making it additionally a good candidate to test whether a low number prior exists.

We predicted that if children possess a low number prior, then they should reliably underestimate the number of objects they see on the side with perceptual noise, choosing it as less numerous. Additionally, we predicted a low number prior would get stronger with age, creating a shift toward adult‐like priors similar to the developmental trajectory of other perceptual priors (although we remain neutral about whether any age‐related changes might occur from experience or maturation).

## 1 Method

### 1.1 Preregistration

This experiment's sample size and primary analyses were preregistered The data, analytic code, and materials necessary to reproduce the analyses presented here are publically accessible. An adult sample was added later to compare children and adults.

### 1.2 Participants

Eighty children (20 per age tested; 39 girls and 41 boys) were recruited from the Vancouver metropolitan area and participated in the study throughout 2022. Sample size was preregistered and was based on past research with similar experimental procedures (Baer and Odic 2020; Halberda and Feigenson 2008). Children were between the ages of 5 and 9 years old (*M* = 6;11 [years;months], range = 5;0–8;11). This age range was based on previous work (Qu et al. 2022) and because pilot testing showed that the task was too long for 4‐year‐old children to reliably finish. Because one of the research questions asked if the low number prior gets stronger with age, children were divided into two age groups: younger children (i.e., forty 5‐ to 6‐year‐olds; *M* = 6;00 [years;months], range = 5;0–6;11) and older children (i.e., forty 7‐ to 8‐year‐olds; *M* = 7;11 [years;months], range = 7;0–8;11). When appropriate, age is treated continuously in analyses.

Six children were excluded and replaced because: (1) they did not complete the entire task (*n* = 4) and (2) the experimenter decided that the child was too distracted or unable to follow instructions during testing (*n* = 2). The pre‐registration included other potential exclusion criteria (e.g., developmental disorders), but none of them were triggered in our sample. To extend the age range further, twenty university students were recruited from the University of British Columbia and participated for course credit. No adult participants were excluded from the pre‐registered criteria.

### 1.3 Materials

**FIGURE 1:**
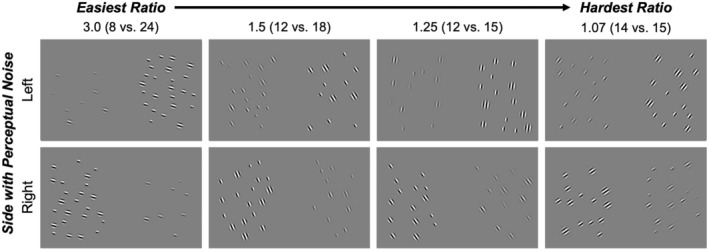
Experimental stimuli. Trials ranged in difficulty, where some were easier to discriminate (e.g., 8 vs. 24) than others (e.g., 14 vs. 15). To force participants to rely on their priors, one of the two sides (e.g., left or right) contained perceptual noise (i.e., low contrast). Additionally, the correct answer (i.e., the more numerous side) was counterbalanced between the two sides. Overall contrast increased on all images for better visibility at the figure's printed size.

To measure number perception, participants completed a number discrimination task where participants report which of two sides contains more items (e.g., “The left side has more dots”). The number discrimination task included displays containing two sets of Gabor patches that were created in MATLAB using custom‐made Psychtoolbox‐3 scripts. Gabor patches were used because such stimuli are typical when adjusting levels of contrast due contrast's relation with spatial frequency (Peli et al. 1996). Each display contained one set of Gabor patches appearing on the left side and the other set appearing on the right side. Trials ranged in difficulty by displaying different ratios between the two sides: 3.0 (8 vs. 24; i.e., least difficult), 1.5 (12 vs. 18), 1.25 (12 vs. 15), and 1.07 (14 vs. 15; i.e., most difficult; see Figure 1). Stimuli were counterbalanced such that both the left and right side contained more objects on half the total trials. To control for the influence of non‐numeric variables (e.g., cumulative area) on number perception, half of trials were considered area congruent trials (i.e., numerically more dots corresponded with a larger cumulative area and equal average dot size compared with the numerically smaller side) and the other half of trials were considered area incongruent trials (i.e., numerically more dots corresponded with a smaller cumulative area and smaller average dot size compared with the numerically smaller side), a method consistent in other studies (Halberda et al. 2008; Odic 2018).

To examine the influence of priors on number perception, perceptual noise was embedded to one side per trial: the left side on 50% of trials and on the right side on the other 50% of trials. Low contrast was defined as reducing contrast to 25%. To make the other side less visually different from the perceptual noise side, contrast was reduced to 80% for those sides. The examples of stimuli are found in Figure 1.

Although we report accuracy as a descriptive variable, the key dependent variable was the proportion of trials in which participants identified the left side as more numerous (i.e., the *bias* for the left side), a method used in previous research (Bonn and Odic 2024; DeWind et al. 2015, 2020; Qu et al. 2022). The left side was chosen arbitrarily. Given that the task was set up so that the left and right side (1) had more dots equally often and (2) contained perceptual noise equally often, any preference toward or away from the left side would indicate if participants were underestimating or overestimating the number of Gabor patches they saw. For example, if participants were consistently underestimating the number of Gabor patches they saw when stimuli was embedded in noise and thus relying on a low number prior, then they should choose the left side as more numerous *less* often when the left side was embedded in perceptual noise. However, when the right side was embedded in perceptual noise, then the left side should be chosen as more numerous *more* often because objects on the right side are being underestimated and pulled toward a low number prior. Following the advice of two anonymous reviewers, we also additionally report the proportion of trials on which the “low perceptual noise” (i.e., high contrast) side was chosen; values above 0.50 indicate a bias consistent with a low number prior. We include this alternative coding of the data to help interpretability for readers unfamiliar with the more traditional side‐bias metric.

### 1.4 Procedures

Participants were either tested online via Zoom or in person in a quiet room on an iMac running PsychoPy. The task included 64 randomly ordered trials divided into 4 blocks. Especially for the younger age group (i.e., 5 years old), completing 64 trials on a number discrimination task is quite demanding, and previous work tends to include only around 32 trials (Baer and Odic 2020; Halberda et al. 2008). To keep child participants engaged and maintain their attention over the entire experiment, an experimenter introduced the experiment to both child and adult participants as a game.

At the beginning of the game, participants were presented with a protagonist who recently stumbled upon some mystery seeds (i.e., the Gabor patches). The Gabor patches were specifically described as “seeds” since Gabor patches are otherwise novel stimuli for children. Participants were told that the protagonist was very excited to see what the seeds turned into, and to help her grow the plants as fast as possible, the participants' job was to help the protagonist figure out which bags (e.g., the left blue bag or the right green bag) had *more* seeds in them.

For the number discrimination trials, participants saw two sets of “seeds” (i.e., Gabor patches), one on the left in a blue rectangle (i.e., a blue bag) and one on the right in a green rectangle (i.e., a green bag) indefinitely. Because online testing meant that we could not precisely control brightness of screens, we made exposure time infinite, allowing children to spend as long as they need encoding the stimuli. Experimenters then pressed the “z” key if participants believed the left side (i.e., blue bag) to have more objects and the “m” key if participants believed the right side (i.e., green bag) to have more objects. If a participant began to count (e.g., verbally, with a pointed hand, or their eye gaze clearly demonstrated counting), the experimenter reminded participants to not count and to “make their best guess.” Experimenters were instructed to not give participants any feedback during the experiment. Instead, experimenters were instructed to motivate participants to finish the task by drawing their attention to the progress bar and reminding participants they were getting closer and closer to seeing the next plant. Each block contained 16 trials with a total of 4 blocks. Adults completed the same exact task as children.

## 2 Results

Several preliminary analyses were conducted to determine whether participants were completing the task similarly to past research. First, we examined participants' overall accuracy across the three age groups. All participants performed well in the task, with adults showing the highest rates of accuracy (*M* = 0.92, SD = 0.28), then older children (*M* = 0.85, SD = 0.36), and younger children demonstrating the lowest rates of accuracy (*M* = 0.83, SD = 0.38). However when we analyzed age as a continuous variable for all the data with children (adults were excluded here since we did not have their exact age), there was no relation between continuous age and accuracy, *r*(78) = 0.14, *p* = 0.209. Additionally, there was no difference between girl's (*M* = 0.84, SD = 0.37) and boy's (*M* = 0.84, SD = 0.37) accuracy scores, *t*(78) = −0.03, *p* = 0.978, *d* = 0.006. There was also no difference between children tested online (*M* = 0.83, SD = 0.38) compared with the children tested in‐person (*M* = 0.86, SD = 0.35), *t*(78) = 1.82, *p* = 0.072, *d* = 0.43. A one‐way repeated measures ANOVA revealed that participants replicated patterns shown in previous work where participants were more accurate on easier ratios like 3.0 (*M* = 0.97, SD = 0.17) compared with harder ratios like 1.07 (*M* = 0.68, SD = 0.47), *F*(3, 297) = 238.10, *p* < 0.001, $\eta_{p}^{2}$ = 0.71, which is expected given Weber's law.

Since non‐numeric variables like cumulative area influence number perception (DeWind et al. 2015; Odic 2018), we compared the rates of accuracy between area congruent (i.e., numerically more dots corresponded with a larger cumulative area) and area incongruent (i.e., numerically more dots corresponded with a smaller cumulative area) trials. The accuracy was impacted by the congruency between cumulative area and number (Congruent: *M* = 0.88, SD = 0.33, Incongruent: *M* = 0.83, SD = 0.38, *t*(198) = 3.63, *p* < 0.001, *d* = 0.51). Such area congruency effects are common when feedback is not given to participants (Dramkin et al. 2022). To further probe the relation between area congruency on overall accuracy, we computed the task difference score by taking the difference in accuracy between area congruent and area incongruent trials and correlated this value with continuous age for all child participants. There was no relation between the task (area congruent, area incongruent) difference score and continuous age, *r*(78) = 0.10, *p* = 0.398, suggesting that this congruency effect did not differ across ages (see also Odic, 2018).

**FIGURE 2:**
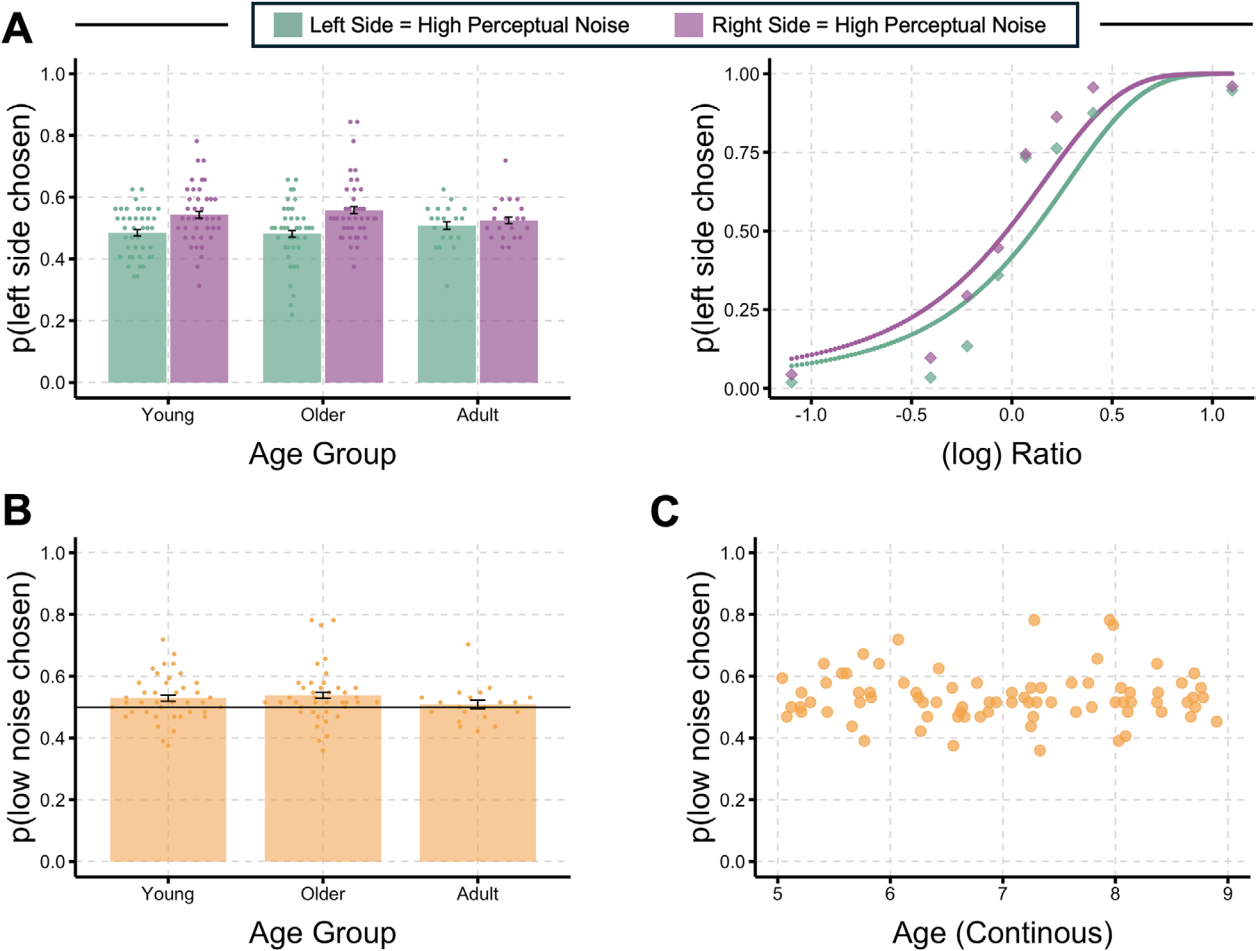
Results. (A) The proportion of left side chosen as the more numerous side across all age groups (left) and ratios (right). If participants are exhibiting a low number prior, then we should expect participants to choose the left side as more numerous *less often* when the left side contains noise but *more often* when the right side contains noise. There should, therefore, be a significant difference between *p*(left side chosen) for the left versus right side noise conditions. Child data was replotted across numerical ratios across all age groups in the right plot. (B) Another way to observe the data is to examine the proportion of participants picking the side with low noise (i.e., high contrast) as more numerous. If participants are exhibiting a low number prior, they should be more likely to choose the side with low noise as more numerous more than 50% of the time. B depicts these results per age group. (C) To examine the child data across ages more closely, the proportion of participants picking the side with low noise (i.e., high contrast) as more numerous is plotted across continuous age.

Next, to address our main research question of whether children and adults demonstrated a low number prior when numerical information was embedded in perceptual noise (i.e., low contrast) we ran the following confirmatory analysis. Figure 2A depicts the proportion of trials that participants selected the left side as more numerous per age group, depending on whether the left or right side contained perceptual noise. Importantly, the correct answer (i.e., the more numerous side) is counterbalanced, meaning that any difference between trials where the left side contains perceptual noise and trials where the right side contains perceptual noise is due to the perceptual noise itself. As shown in the left panel of Figure 2A, when the left side contains perceptual noise (green bars), participants are *less* likely to indicate the left side as more numerous. In other words, they are underestimating the number of perceptually noisy Gabor patches. Alternatively, when the right side contains perceptual noise (purple bars), participants are *more* likely to indicate the left side as more numerous. The right panel of Figure 2A shows the effect across numerical ratios for the child data. The separation in the curves demonstrates that perceptual noise resulted in underestimation across ratios. Again, when the left side contains perceptual noise, the left side was perceived as less numerous across ratios and chosen *less* often, whereas when the right side contained perceptual noise, the right side was perceived as less numerous, resulting in the left side being chosen *more* often.

To quantify this underestimation for the side with perceptual noise, we ran a 2 (Perceptual Noise Side: left, right) by 3 (Age Group: younger, older, adult) on proportion of trials where the left side was chosen as more numerous. Analyses revealed a main effect of Perceptual Noise Side, *F*(1, 97) = 13.20, *p* < 0.001, $\eta_{p}^{2}$ = 0.12. Such medium effect sizes are consistent with previous number comparison work with children and adults that create perceptual noise through stimulus entropy (Qu et al. 2022; DeWind et al. 2020). There was no main effect of Age Group, *F*(2, 97) = 0.15, *p* = 0.859, $\eta_{p}^{2}$ = 0.003. Additionally, there was no significant interaction between Perceptual Noise Side and Age Group, *F*(2, 97) = 0.95, *p* = 0.390, $\eta_{p}^{2}$ = 0.02. All mean values and standard deviations are found in Table 1. Overall, these results suggest that all age groups underestimated the number of Gabor patches on the side embedded in noise, consistent with a lower number prior. The lack of differences between age groups suggests that this low number prior does not get stronger over the age range tested.

**TABLE 1:** Proportion of trials where the left side was indicated as more numerous.

Age group	Left side = perceptual noise		Right side = perceptual noise	
	*M*	SD	*M*	SD
Younger	0.49	0.08	0.54	0.10
Older	0.48	0.10	0.56	0.10
Adults	0.51	0.07	0.53	0.07

*Note:* Perceptual noise was defined as low contrast. Given the hypothesis, if participants exhibit a low number prior, then it is expected that participants choose the left side as more numerous *less often* when the left side contains noise but *more often* when the right side contains noise.

To further demonstrate this underestimation effect in a more intuitive way, we plotted the proportion of trials where the low perceptual noise (i.e., high contrast) side was chosen as more numerous across age (Figure 2B) and used this dependent variable in the following exploratory analyses. We examined age as a continuous factor for all child data (Figure 2C). There was no relation between the proportion of trials where the low perceptual noise side was chosen as more numerous and continuous age, *r*(78) = 0.004, *p* = 0.971, suggesting that this underestimation effect did not differ across age, even when treated continuously.

Previous work has shown that exposure time can influence estimation. For example, longer exposure time tends to lead to estimates being less noisy (Inglis and Gilmore 2013) and less biased (Cheyette and Piantadosi 2019). To check if leaving stimuli up indefinitely throughout the experiment influenced the results of this study, we ran an exploratory correlation between (log) reaction time and the proportion of trials where the low perceptual noise was chosen as more numerous for all child data. There was no relation between (log) reaction time and choosing the side with low perceptual noise as more numerous, *r*(78) = 0.07, *p* =0.519. We suspect that this correlation would have occurred, however, if stimuli were presented for significantly less time (i.e., < 100 ms), consistent with previous work.

Another way in which exposure time can influence estimation is by allowing participants to count, rather than perceptually estimate, the number of dots. Beyond not allowing children to count whenever they attempted to do so explicitly in the task, the RT data also strongly suggests that children were not subvocally counting. The typical speed at which young children count is around 0.55–1.00 s/item (Svenson and Sjöberg 1978; Trick et al. 1996). In our task, the number of dots ranged from 27 to 32 depending on ratio, which would imply that average RTs should be between 14.8 and 17.6 s for all trials. Instead, we find that the median RT across all trials is 4.78 s, far too fast for children to be subvocally counting.

**FIGURE 3:**
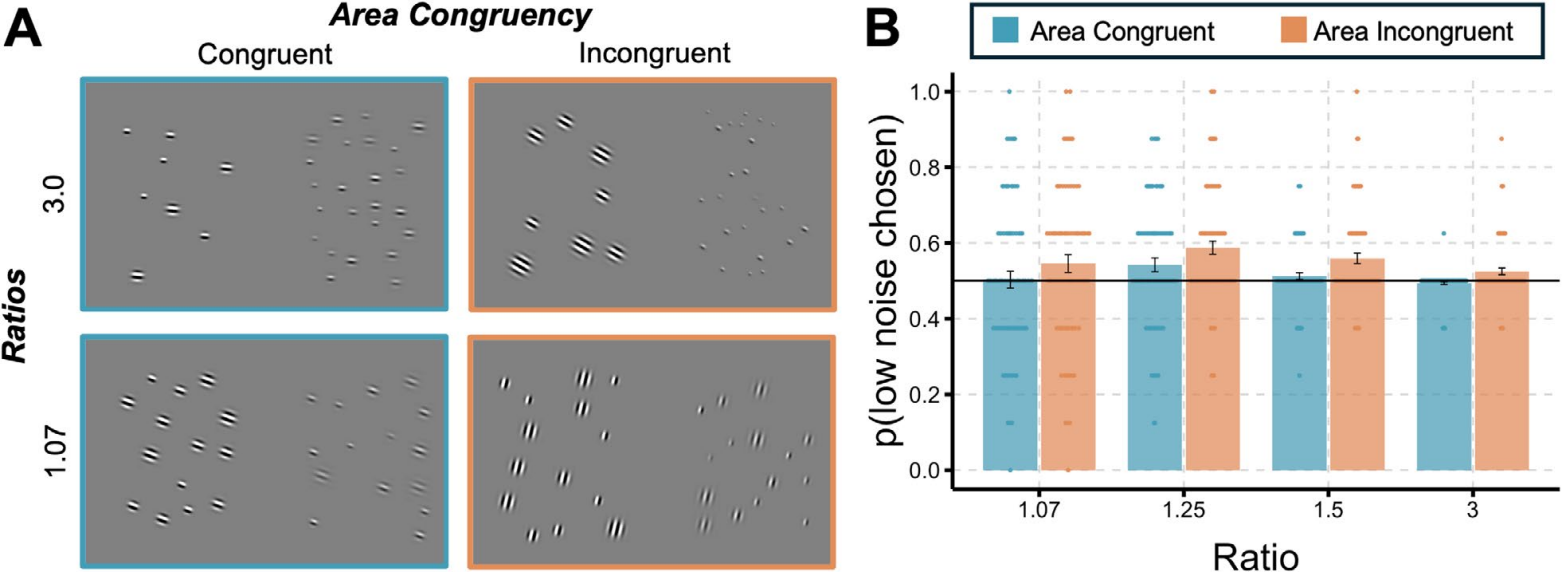
(A) Example of area congruent and area incongruent trials for the largest ratio (3.0) and the smallest ratio (1.07). In both examples, the right side contains both high perceptual noise (i.e., low contrast) and the more numerous value (i.e., 24, 15), although analyses were conducted on all trials irrespective of which side contained noise or the more numerous value. As can be seen in the area incongruent trials, the side with more Gabor patches must take up *less* area than the side with less Gabor patches, making some of the dots on the right side particularly difficult to see, especially for the 3.0 ratio. This is made even more difficult by the addition of high perceptual noise. For the 1.07 ratio, the difference in Gabor patch sizes between the left and right side is negligible. If participants are missing Gabors during encoding, then there should no difference in responses between area congruent and area incongruent trials for smaller ratios like 1.07 given that the Gabor size is similar between area congruent and incongruent trials. However, for larger ratios like 3.0 we should see greater underestimation for area incongruent trials compared with area congruent trials if participants are missing Gabors during encoding. Overall contrast increased on all images for visibility. (B) The proportion of trials in which children picked the low perceptual noise side as more numerous across ratios and area congruency (congruent, incongruent). If participants were merely missing Gabor patches during encoding, then we would expect the greatest difference in *p*(low noise chosen) between area congruent and area incongruent trials for the 3.0 ratio and the smallest difference for the 1.07 ratio. Instead, we find similar patterns across all ratios. Adults demonstrate a similar pattern to children.

One important alternative explanation of our findings is that the stimuli with higher perceptual noise were less visible and easily missed when encoding the visual scene, leading to underestimation (or, alternatively, that low perceptual noise stimuli were encoded better, leading to overestimation). Previous work has shown that low contrast can sometimes *increase* the perceived number of objects, as participants might confuse the background for the presence of more objects (Lei and Reeves 2023). Nevertheless, to investigate this concern, we relied on the area congruency manipulation, as area incongruent trials have Gabor patches that are more numerous but are also significantly *smaller* (and therefore especially easily to miss during encoding). This is especially the case for our larger ratios (3.0) compared with smaller ratios in area incongruent trials (1.07; see Figure 3A for examples): on a ratio 3.0, the more numerous set has dots that are a *third* of the total cumulative area compared with the less numerous side, whereas on a ratio of 1.07 they are only 7% smaller (a value that is nearly indistinguishable given typical area perception Weber fractions; Odic 2018). Adding perceptual noise, where these already small dots are now corrupted by low contrast, would make dots on these trials even more susceptible to being missed during encoding. Therefore, if the observed effect was primarily driven by the participants simply not seeing the low contrast and highly noisy dots, we should expect a strong interaction between area congruency and ratio: area incongruent high ratios should show more underestimation compared with area congruent trials, whereas area incongruent low ratios should show a similar bias to area congruent trials. A 2 (area congruency: congruent, incongruent) by 4 (ratio: 3.0, 1.5, 1.25, and 1.07) repeated measures ANOVA over the proportion of trials in which low perceptual noise was chosen as more numerous for all child data revealed a main effect of area congruency, *F*(1, 79) = 21.69, *p* < 0.001, $\eta_{p}^{2}$ = 0.22, and a main effect of ratio, *F*(3, 237) = 4.64, *p* = 0.004, $\eta_{p}^{2}$ = 0.06. However, most importantly, there was no significant interaction between area congruency and ratio, *F*(3, 237) = 0.15, *p* = 0.93, $\eta_{p}^{2}$ = 0.001. As shown in Figure 3, the effect was almost the reverse of what was expected: the trials with the smallest low‐contrast dots show the *least* bias, whereas the trials with the larger ones produce more. Including the adults' data in this analysis produced similar results. This suggests that participants did not show a stronger underestimation bias on trials where the Gabor patches were easier to miss, likely because the infinite display time allowed for as much scanning time as participants needed to encode the stimuli.

## 3 Discussion

If the perceptual representations of number are tuned to real‐world scene statistics, then we would expect the presence of a low number prior: a general expectation that low numbers occur in the environment more often than high numbers. We provide a test of this hypothesis by examining if children's and adults' number estimation is skewed toward low numbers in situations where the sensory input is partly corrupted by noise. Although there are cases of prior repulsion when there is sensory (internal) noise, we specifically utilized stimulus (external) noise and thus expected participants to be biased toward a low number prior (Wei and Stocker 2015). All age groups were less likely to pick the side with perceptual noise as the more numerous side (i.e., participants were underestimating the number of objects when the side included high perceptual noise), thus demonstrating a low number prior. The low number prior did not differ between age groups, suggesting that the prior is developed by 5 years old and does not change across the age range tested (unlike, e.g., the light‐from‐above prior; Stone 2011). Although the effect was numerically (but not significantly) smaller in adults, this probably stems from their better ANS precision and stronger contrast sensitivity making the manipulation slightly weaker (Beazley et al. 1980; Odic 2018).

Why might children possess number‐related priors? The ANS may have adapted evolutionarily to include this low number prior to move more optimally through a complex visual world where smaller numbers (e.g., 10 cookies, 6 chairs, etc.) are more likely to be encountered compared with larger numbers (e.g., 1000 bees). Alternatively, a low number prior might instead emerge from our daily experience interacting with lower numbers and develop as we gain more and more experience viewing objects in our world. However, if this was the case, one might predict that older participants should have demonstrated a *stronger* low number prior, which we did not observe. This could indicate that (1) the ANS low number prior does not emerge via gradual experience and may instead be an innate prior adapted to optimally navigate our world; (2) the prior emerges via gradual experience but is fully developed by 5 years old; or (3) our task was not sensitive enough to detect a small developmental improvement that occurs after age 5. Given the current study, we cannot say for certain which of these is most probable. To disentangle these accounts of the ANS and to determine how this low number prior emerges, a younger age range should be tested in future work to investigate whether differences in the low number prior exist across development.

Qu et al. (2022) provides convergent evidence that humans may utilize a low number prior. They demonstrate that participants tend to underestimate numbers when displays are high in entropy or include incoherent elements (e.g., heterogenous orientations or colors), compared with the displays that are low in entropy (e.g., homogenous orientations or colors). The authors note that one (though not sole) explanation for this effect may be that participants are influenced by prior distributions of numbers. Like low contrast, high entropy may potentially result in less information being available from the sensory input alone, forcing participants to rely more heavily on a low number prior. In their work, however, the effect of entropy grows over development, suggesting either that low number priors are used differently for different types of noise (i.e., contrast vs. entropy) or that the explanation for their effect is not based in the usage of low number priors, but instead on, for example, how well children choose relevant objects of enumeration in high‐entropy displays.

The current work contributes to not only the possession of number‐related priors in children and adults but also the integration of such priors into their perception. Previous work shows that not all individuals who possess a prior consistently use it. For example, even if it would better their performance on a visual serial comparison task, individuals with dyslexia show difficulties integrating priors based on distributional information from their environment as opposed to matched controls (Jaffe‐Dax et al. 2016). Future work should further explore when exactly this integration takes place for some individuals and not others, and whether it can explain any individual differences in ANS perception (e.g., for children with and without dyscalculia; Piazza et al. 2010).

The idea that people are sensitive to the statistics of their environment is not new. There has been work demonstrating infants' and young children's sensitivity to an experiment's local statistics that can bias numeric perception, whether that be through calibration (Izard and Dehaene 2008) or the familiarization of prior numeric information in a task resulting in hysteresis (Odic et al. 2014; Wang et al. 2018). The current experiment extends this work on local statistics and the influence of prior information at shorter time scales to the statistics potentially learned over a lifetime. Additionally, unlike in the case of hysteresis, participants in our task did not receive any feedback, meaning that participants utilized a low number prior, and thus long‐term environmental statistics, without explicit knowledge that this would benefit them on the task.

## 4 Conclusion

The current research is the first to experimentally test for a low number prior in both children and adults. Although previous work suggests that we may have a low number prior given the natural distribution of objects in our world (Testolin et al. 2020; Piantadosi 2016; Sanford and Halberda 2024), our research is the first to empirically test the presence of a low number prior in human observers and test this prior's developmental trajectory. Although current study cannot say for certain if this low number prior emerges via experience or maturation, this work demonstrates ANS's sensitivity to real‐world statistics, including a negative power‐law distribution of objects typical in natural scenes.

## Author Contributions

M.N.L. performed conceptualization, data curation, formal analysis, investigation, methodology, validation, visualization, writing original draft, and writing review in editing. In addition to all of the above roles, D.O. also performed funding acquisition, project administration, resources, software, and supervision.

## Disclosure

Our study shows children have implicit knowledge that most objects in the world obey a “low number distribution”: smaller numbers of things (e.g., three pencils) occur much more often than larger numbers of things (e.g., three thousand pencils). This “prior” is present in children as young as five and does not strengthen with age. Children's intuitive number sense is, therefore, sensitive to the statistical information in the real‐world.

## Data Availability

The data necessary to reproduce the analyses presented here are publicly accessible. Data are available at the following URL: https://osf.io/e2r8x/files/osfstorage. The analytic code necessary to reproduce the analyses presented in this paper is publicly accessible. Analytic code available at the following URL: https://osf.io/e2r8x/files/osfstorage. The materials necessary to attempt to replicate the findings presented here are publicly accessible. Materials available at the following URL: https://osf.io/e2r8x/files/osfstorage. The analyses presented here were preregistered. The preregistration is available at the following URL: https://osf.io/t8jau
